# Autosomal Recessive Rod-Cone Dystrophy Associated With Compound Heterozygous Variants in *ARL3* Gene

**DOI:** 10.3389/fcell.2021.635424

**Published:** 2021-03-04

**Authors:** Leming Fu, Ya Li, Shun Yao, Qingge Guo, Ya You, Xianjun Zhu, Bo Lei

**Affiliations:** ^1^Henan University People's Hospital, Henan Provincial People's Hospital, Zhengzhou, China; ^2^Henan Branch of National Clinical Research Center for Ocular Diseases, Henan Eye Institute/Henan Eye Hospital, People's Hospital of Zhengzhou University, Henan Provincial People's Hospital, Zhengzhou, China; ^3^Department of Laboratory Medicine, Sichuan Provincial People's Hospital, School of Medicine, University of Electronic Science and Technology of China, Chengdu, China

**Keywords:** *ARL3*, compound heterozygous variants, rod-cone dystrophy, cone-rod dystrophy, RP2

## Abstract

**Purpose:** *ARL3* (ADP-ribosylation factor-like 3) variants cause autosomal dominant retinitis pigmentosa (RP) or autosomal recessive Joubert syndrome. We found a family with rod-cone dystrophy (RCD) and verified it was associated with compound heterozygous variants in *ARL3* gene.

**Methods:** Ophthalmic examinations including optical coherence tomography and electroretinogram (ERG) were performed. Targeted next generation sequencing (NGS) was performed for the proband using a custom designed panel. Sanger sequencing and co-segregation were conducted in the family members. Changes of protein structure mediated by the variants were studied *in vitro*. ARL3 protein stability and its interaction with RP2 protein were assessed by cycloheximide chase assay and co-immunoprecipitation (Co-IP) assay.

**Results:** Visual acuity of the 18-year-old male proband was 0.25 in the right and 0.20 in the left eye, while his non-consanguineous parents and sister was normal. The proband showed signs of RCD, including nyctalopia, peripheral field loss, bone-spicule deposits in the retina, and reduced ERG responses. The father, aged 50 years old, showed visual acuity of 1.0 in both eyes. Unlike the proband, he presented late onset and mild cone-rod dystrophy (CRD), including macular atrophy, central scotomata, moderate reduction in photopic ERG responses. None of all the family members had hearing abnormality, mental dysplasia or gait instability. We identified two novel compound heterozygous variants (c.91A>G, p.T31A; c.353G>T, p.C118F) in *ARL3* in the proband, while his father only had variant c.91A>G. Bioinformatics analysis indicated amino acid positions of the two variants are highly conserved among species. The *in silico* tools predicted the variants to be harmful. Protein structure analysis showed the two variants had potential to alter the protein structure. Based on the ACMG guidelines, the two variants were likely pathogenic. In addition, the *ARL3* mutations destabilized ARL3 protein, and the mutation c.353G>T disrupted the interaction between ARL3 and RP2 in HEK293T cells.

**Conclusions:** We showed novel compound heterozygous variants in *ARL3* were associated with early onset of autosomal recessive RCD, while c.91A>G along may be associated with a late onset of dominant CRD. The two variants in *ARL3* could be causative by destabilizing ARL3 protein and impairing its interaction with RP2 protein.

## Introduction

Retinitis pigmentosa (RP) is a group of highly heterogeneous inherited retinal diseases, and it is one of the most important causes of blindness worldwide (Narayan et al., 2016). Almost 2.5 million individuals suffered with RP around the world (Hu et al., 2019). RP is typically characterized by night blindness (nyctalopia), progressive constriction of visual field, changes in the fundus and reduced electroretinogram (ERG), and ultimately resulting in complete blindness (Ali et al., 2017). Rod-cone dystrophy (RCD) is a common form of RP due to primary degeneration of rod cells followed by degeneration of cone cells (Pagon, 1988). RP manifests in a syndromic or a non-syndromic form. Non-syndromic RP can be in different traits, including autosomal dominant RP (adRP), autosomal recessive RP (arRP), X-linked recessive form and simplex/sporadic type. Nowadays, around 90 genes were identified to be associated with non-syndromic RP (https://sph.uth.tmc.edu/retnet/). The proteins encoded by RP-associated genes exert different roles in transcription, retina phototransduction, transport processes via the photoreceptor connecting cilium, cell growth, cellular structure, and metabolism of vitamin A (Collin et al., 2011; Bhatia et al., 2019).

Human *ARL3* (ADP-ribosylation factor-like 3; MIM:604695) gene is mapped to chromosome 10q24.32, and contains 6 exons. The encoded protein ARL3 is a small molecule GTP-binding protein belonging to the ADP-ribosylation factor (ARF) family. In the mammalian retina, ARL3 is mainly localized to microtubule structures throughout the retina and is enriched in the connecting cilium of rod and cone photoreceptors (Grayson et al., 2002). ARL3 is crucial for ciliogenesis and axoneme formation, as well as cargo displacement of lipidated proteins in the cilium (Hanke-Gogokhia et al., 2016). Besides, ARL3 also acts as an allosteric factor for the release of lipidated proteins bound to PDE6D (delta subunit of phosphodiesterase) and UNC119A/B in an apparent GTP dependent manner (Hanke-Gogokhia et al., 2018). The activity of ARL3 is regulated by a GTP-exchange factor ARL13B (ADP-ribosylation factor-like protein 13B) (Gotthardt et al., 2015) and a GTPase-activating protein RP2 (Retinitis Pigmentosa 2) (Veltel et al., 2008).

A missense variant in *ARL3* (NM_004311.3) (c.269A>G, p.Tyr90Cys) has previously been reported as a possible cause of non-syndromic autosomal dominant RP in two families (Strom et al., 2016; Holtan et al., 2019). In addition, homozygocity for two different *ARL3* Arg149 missense variants (c.445C>T, p.Arg149Cys; c.446G>A, p.Arg149His) were reported to cause Joubert syndrome (Alkanderi et al., 2018). A most recent report indicated that a homozygous variant in *ARL3* (c.296G>T, p. Arg99Ile) caused cone-rod dystrophy (CRD) in two consanguineous families (Sheikh et al., 2019). In this study, we found a Chinese family with typical rod-cone dystrophy (RCD), which was associated with novel compound heterozygous variants in *ARL3*. In addition, we presumed that one of the variants might be associated with a late onset of dominant CRD. This study expanded both the phenotype and genotype of *ARL3* associated retinal dystrophy. Furthermore, our data suggested that the pathogenicity of the variants were related with destabilizing of ARL3 protein, as well as impairing its interaction with RP2, another protein that is associated with RP.

## Materials and Methods

### Subjects and Clinical Assessment

The study was performed in accordance with the Declaration of Helsinki and approved by the Ethics Committee of Henan Eye Hospital for the release of clinical information, family history, and blood extraction for genetic testing [IRB approval number: HNEECKY-2019 (15)]. Written informed consent was obtained from all participants after the study risks and benefits were thoroughly explained.

All the members were enquired about the family and medical history. Each member was accepted a complete ocular examination, including best corrected visual acuity (BCVA), intraocular pressure (IOP), slit-lamp biomicroscopy, color vision, fundus photography, visual field, swept-source optical coherence tomography (SS-OCT, VG200D SVision Imaging, China), and full-field electroretinogram (ERG).

### Targeted Gene Sequencing and Data Analysis

Genomic DNA was extracted from peripheral blood of all family members with the TIANGEN Blood DNA Kit (DP304, TIANGEN, China). Targeted next generation sequencing (NGS) was performed for the proband using a custom designed panel (PS400) containing 376 known genes associated with inherited retinal diseases (Zhu et al., 2021). The Nextseq 500 (Illumina, San Diego, CA, USA) platform was used for paired-end sequencing with read lengths of 150 bp and average sequencing depth of almost 300 X. Raw reads were mapped to the human genome reference (UCSC hg19) using three commercial software including XYGeneRanger 2.0 (Xunyin, Shanghai, China), TGex (LifeMap Sciences, Alameda, CA, USA) and Efficient Genosome Intepration System, EGIS (SierraVast Bio-Medical Technology Co., Ltd, Shanghai, China). Variant-filtering was based on public and in-house SNP databases, including 1000Genome project, HGMD, ExAC and ClinVar, as well as our internal database. The non-synonymous and splicing variants with MAF <2% were kept for further analysis. Sanger sequencing and co-segregation analysis were performed for the verification of suspicious disease-relevant gene variants in the available family members. Primer sequences and PCR conditions were shown in Supplementary Tables 1, 2, respectively.

### *In silico* Molecular Genetic Analysis and Bioinformatics Analysis

To assess the possible pathogenicity of these mutations, and to predict whether a protein sequence variation affected protein function, the following web applications was used: LRT (Likelihood Ratio Test), PolyPhen-2 (Polymorphism Phenotyping v2), Mutation Taster (Schwarz et al., 2010), SIFT (Sorting Intolerant From Tolerant) (Kumar et al., 2009), FATHMM (Functional Analysis Through Hidden Markov Models) (Rogers et al., 2018), and CADD (Combined Annotation Dependent Depletion) (Kircher et al., 2014). Multiple protein sequence alignment among various species was carried out by Clustal Omega (Sievers and Higgins, 2018). The tertiary structure of protein was predicted by the Swiss-Model workspace (http://swissmodel.expasy.org) (Biasini et al., 2014). CDD online software (https://www.ncbi.nlm.nih.gov/Structure/cdd/wrpsb.cgi) was used to conduct domains analysis. HOPE online software (http://www.cmbi.umcn.nl/hope) was used to analyze the structural effects of mutation. Superimposition of homology models of ARL3 was constructed using PyMOL software (https://pymol.org/2/). The protein stability of *ARL3* mutations was predicted with the online tools MUpro (http://mupro.proteomics.ics.uci.edu/) (Cheng et al., 2006)and I-Mutant v2.0 (http://folding.biofold.org/i-mutant/i-mutant2.0) (Capriotti et al., 2005). All the sources were provided in the public domain.

### Cell Culture

HEK293T cell line was purchased from American Type Culture Collection. Cells were cultured in DMEM/HIGH GLUCOSE medium (SH30022.01, Hyclone, USA) containing 10% fetal bovine serum (35-081-CV, Corning, USA) with 100 units/mL penicillin and 100 μg/mL streptomycin at 37°C with 5% CO_2_.

### Plasmids and Transfection

The wild-type, c.91 A>G (p.T31A), and c.353G>T (p.C118F) CDS sequences of the *ARL3* gene with the Flag tag at the C-terminal were synthesized and subcloned into the pcDNA3.1 (+) expression vector (Invitrogen, USA), respectively. The wild-type *RP2* cDNA with the HA tag at the N-terminal was subcloned into the pcDNA3.1 (+) expression vector. All plasmids were confirmed by DNA sequencing and WB analysis. When cells grow to 80–90% confluence, cell transfection was performed using Lipofectamine 3000 (L3000015, Invitrogen, USA). After transfection, the cells were harvested at 24 h for protein extraction and further analysis.

### Western Blots and Antibodies

The primary antibodies used were as follows: the flag antibody (147935, CST, USA), β-actin antibody (200068-8F10, ZENBIO, China), RP2 antibody (ab174840, Abcam, UK). Cells were lysed by RIPA lysis buffer (PC101, EpiZyme, China) with 1% protease inhibitor cocktail (GRF101, EpiZyme, China). The samples were resolved in 12% SDS-PAGE gel and transferred to a polyvinylidene difluoride (PVDF) membrane (IPVH00010, MILLIPORE, Ireland). The membranes were blocked with 5% non-fat dry milk for 1 h and then incubated with the primary antibody overnight at 4°C. After three washes with TBST (containing 1% Tween-20), the membranes were treated with horseradish peroxidase-conjugated secondary antibody at room temperature for 2 h. Then the membranes were exposed to X-ray film after washed for three times.

### Protein Stability Assay

Protein stability was assessed by cycloheximide chase assay. Cycloheximide (CHX, 66-81-9, Selleck, USA), a protein synthesis inhibitor, was used. HEK293T cells were transfected with empty vector, wild-type, T31A and C118F mutation *ARL3* vectors for 24 h. The cells were subsequently treated with 100 μg/ml CHX for 1, 3, and 6 h, respectively. The total protein was extracted for Western blot. Wild-type and mutant ARL3 protein levels were detected using flag antibody. The protein levels of β-actin were the endogenous control.

### Co-immunoprecipitation (Co-IP) Assay

HEK293T cells were co-transfected with 6.25 μg of HA-RP2-WT plasmid and 6.25 μg of either wild-type or mutant Flag-ARL3 plasmids, and harvested for protein extraction after 24 h. Total protein lysate was extracted by immunoprecipitation buffer (BL509A, Biosharp, China), and the concentration of the supernatants was quantified with a BCA protein assay kit (P0011, Beyotime, China). Total proteins of 300 μg were added with 5 × SDS to prepare as the input sample. Total proteins of 500 μg were mixed with 10 μg anti-Flag magnetic beads (HY-K0207, MCE, USA) and shaken for 4 h at 4°C on rotor. The beads were collected by Magnetic Separator and washed three times by immunoprecipitation buffer with 1% protease inhibitor. The beads were mixed with sample loading buffer (1 ×) and boiled for 10 min. The supernatant was collected and used for western blot analysis.

### Statistical Analysis

We normalized the data from each group by dividing the measurement of the tested variant from that of the references. The value of the control was set to one and tested measurement was adjusted accordingly. GraphPad Prism 8.0 was used for the statistical analysis. Student's *t*-test and one-way analysis of variance (ANOVA) were performed with a 95% confidence level to evaluate the differences. *P*-value < 0.05 was considered statistically significant. All quantitative data were displayed as mean ± standard deviation (SD).

## Results

### Clinical Features

**Figure 1:**
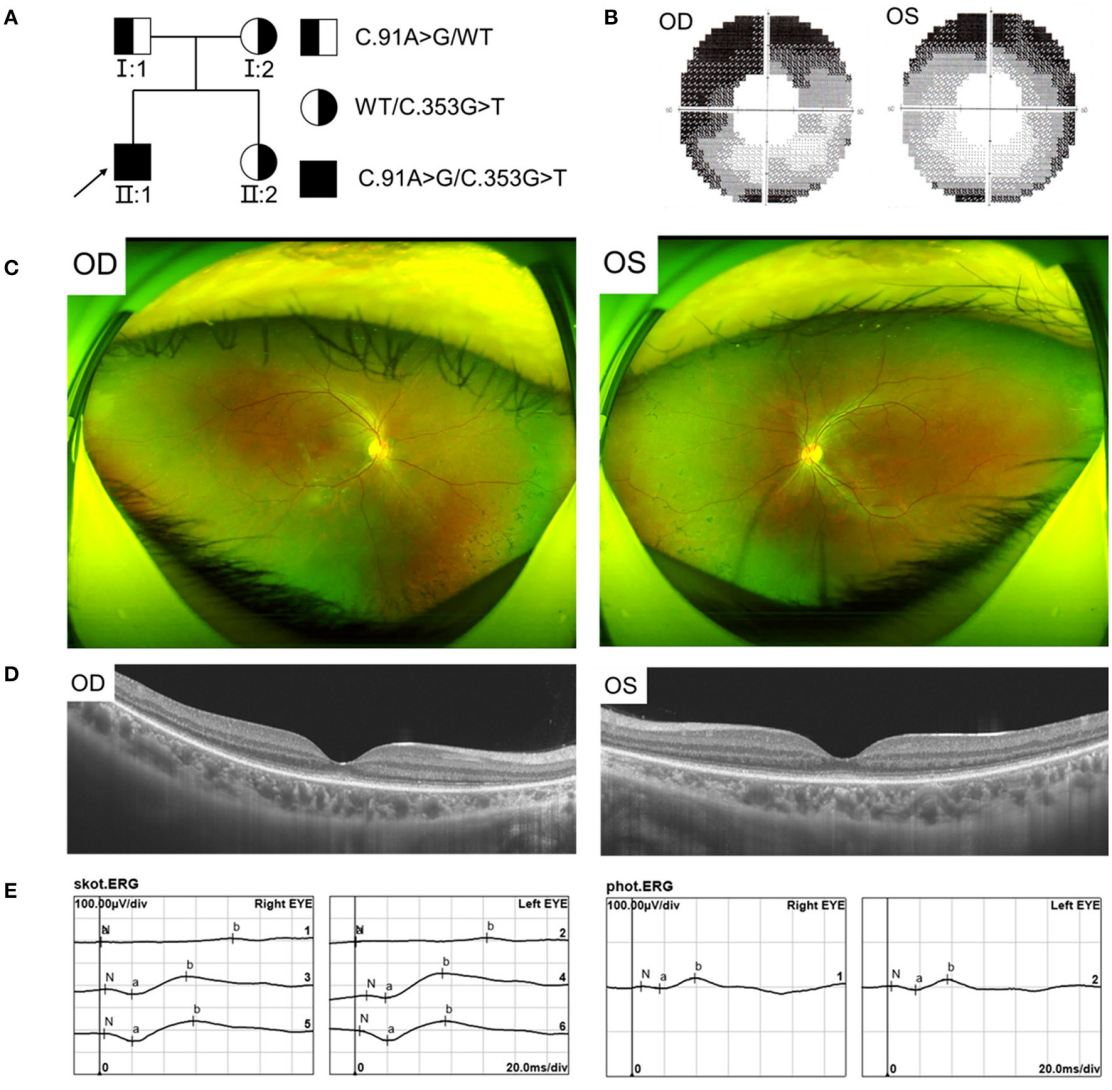
Pedigree and Clinical features of the proband with RCD. **(A)** Pedigree of the family with autosomal recessive RCD. **(B)** Visual field showed tunnel vision. **(C)** Fundus photographs showed peripheral bone spicule pigmentation. **(D)** SS-OCT showed atrophy of the retinal outer layers of the binocular macular area. **(E)** ERG showed severe reduced rod responses and to less extent cone responses.

We found a Chinese family with RCD, the pedigree was presented in Figure 1A. In this family, the proband showed typical RCD but his father present with a late onset and mild CRD, while the mother and sister were unaffected. The proband, a 19-year-old male, had decrease of vision and nyctalopia for 4 years. The BCVA was 0.25 in the right and 0.20 in the left eye. The proband also had dyserythrochloropsia. Visual field testing revealed tunnel visual field (Figure 1B). Fundus photography showed peripheral bone spicule pigmentation (Figure 1C). SS-OCT presented atrophy of the retinal outer layers of the binocular macular area where the light reflection signal of ellipsoid zone and chimeric zone was weakened, coarse and interrupted. The reflection of RPE/Bruch membrane was rough (Figure 1D). ERG showed severe reduction in scotopic ERG responses and to a less extent in photopic ERG responses (Figure 1E).

**Figure 2:**
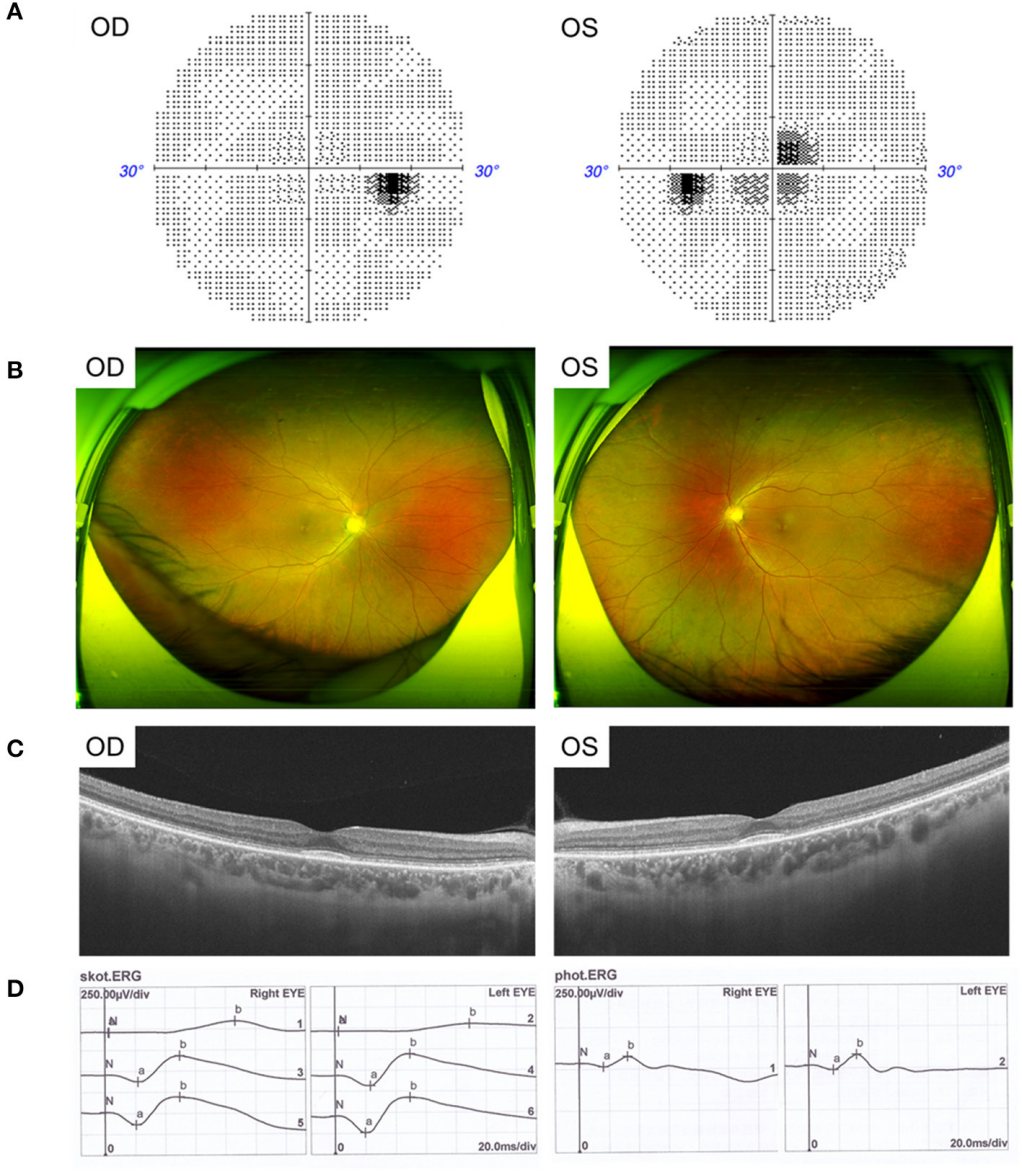
Clinical features of the father with CRD. **(A)** Visual field showed central scotoma. **(B)** Fundus photographs showed circular degeneration around macular fovea. **(C)** SS-OCT revealed the binocular macular area outer retinal was thinned by atrophy. **(D)** ERG showed a moderate decrease in the binocular cone and rod systems.

The father, aged 50 years old, showed normal visual acuity of 1.0 in both eyes and had mild dyserythrochloropsia. Unlike the proband, the father's vision field appeared as central scotoma (Figure 2A). Fundus examination revealed circular degeneration around macular fovea without pigmentation of the peripheral retina (Figure 2B). SS-OCT revealed the binocular outer retinal layers of the macular area was thinned with macular sparing, and the light reflection signal of the ellipsoid zone in the parafovea was weakened and discontinuous (Figure 2C). The ERG showed a moderate decrease in the binocular cone and rod systems (Figure 2D).

Other examinations including anterior segment and IOP of the proband and his father were normal. Neither the proband's mother nor sister showed any abnormalities in the eyes. None of all the family members, including the proband, presented hearing abnormality, mental dysplasia, or gait instability.

### Two Novel Missense Variants in *ARL3* Were Identified

**Figure 3:**
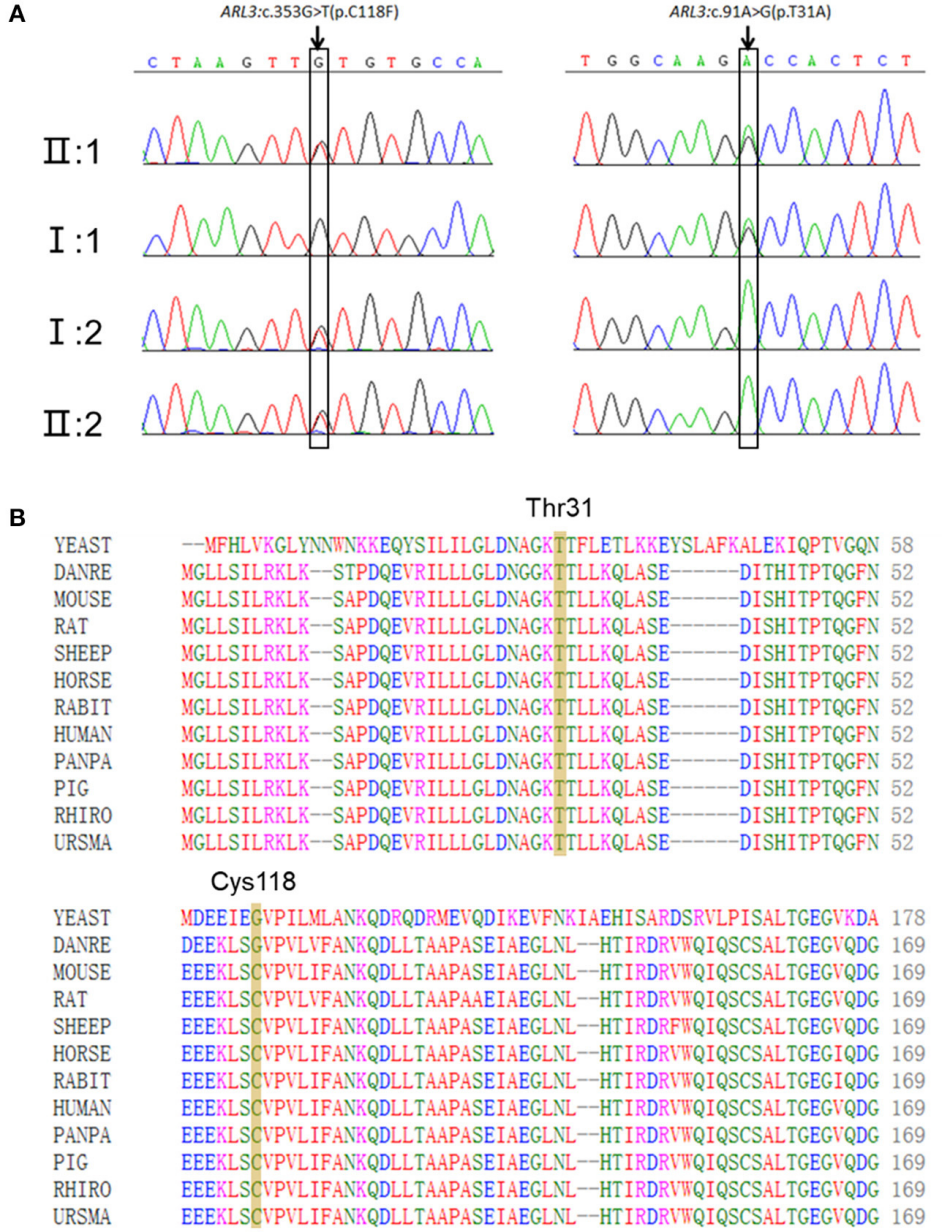
Two novel *ARL3* variants (c.91A>G, p.T31A; c.353G>T, p.C118F) were identified in the RCD family. **(A)** Sanger sequencing in all family numbers. **(B)** Multiple alignments of Thr31 and Cys118 of ARL3 protein from different species. Both variants occurred on the conserved residues of the ARL3 protein.

Targeted NGS identified two novel compound heterozygous variants (c.91A>G, p.T31A; c.353G>T, p.C118F) in *ARL3* in the proband. Sanger sequencing of *ARL3* revealed segregation of the two variants with the RCD phenotype in participating individuals (Figure 3A). Neither variant had been reported in the human gene mutation database (HGMD). The reported allele frequency for c.353G>T (p.C118F) was concentrated in the East Asian populations almost 0.0010 in the Genome Aggregation Database (GnomAD), while no record for the c.91A>G (p.T31A) mutation was found. Furthermore, c.353G>T (p.C118F) has not been reported homozygous in GnomAD. Multiple alignments of amino acid sequences of ARL3 protein from different species revealed that Thr31 is evolutionally conserved among species, while Cys118 is only conserved in mammals, but not in zebrafish and yeast (Figure 3B). Moreover, both of the variants T31A and C118F were considered “Deleterious” as predicted by LRT, Mutation Taster, SIFT, FATHMM, and CADD, but Polyphen-2 predicted the pathogenicity for the variant C118F benign (Table 1).

**Table 1:** *In silico* pathogenicity prediction.

**Algorithm**	***ARL3*: c.91A>G (p.T31A)**	***ARL3*: c.353G>T (p.C118F)**
LRT	0 (Deleterious)	0 (Deleterious)
PolyPhen-2_HDIV	0.99 (Probably damaging)	0.105 (Benign)
PolyPhen-2_HVAR	0.756 (Probably damaging)	0.125 (Benign)
Mutation Taster	1 (Disease-causing)	0.998 (Disease-causing)
SIFT	0.023 (Damaging)	0 (Damaging)
FATHMM	−2.34 (Damaging)	−1.55 (Damaging)
CADD	26.8 (Damaging)	25.4 (Damaging)

*Results for the variants c.91A>G (p.T31A) and c.353G>T (p.C118F) in ARL3*.

In addition, other variants in the proband were screened out, including two heterozygous missense variants (c.13491T>A, p.F4497L; c.11197A>G, p.N3733D) in *USH2A* and a heterozygous missense variant (c.2432T>C, p.I811T) in *FBN2* (Supplementary Table 3), all of which were inherited from his mother (Supplementary Figure 1). These two mutations in *USH2A* were cis-mutations, and the results predicted by *in silico* prediction such as SIFT, Polyphen-2, Mutation Taster, and CADD indicated that these two missense mutations might be benign. Moreover, neither the proband nor his mother showed sensorineural deafness. On the other hand, we noticed that the variant c.2432T>C in *FBN2* was predicted to be possibly damaging by *in silico* prediction. Variants in *FBN2* have been reported to associated with congenital contractural arachnodactyly (CCA) (Putnam et al., 1995) and early-onset macular degeneration (MD) (Ratnapriya et al., 2014), whereas the proband's mother and sister who carried the heterozygous missense variant (c.2432T>C, p.I811T) in *FBN2* didn't have any phenotypes of CCA or MD.

### Protein Structure and Function Prediction

**Figure 4:**
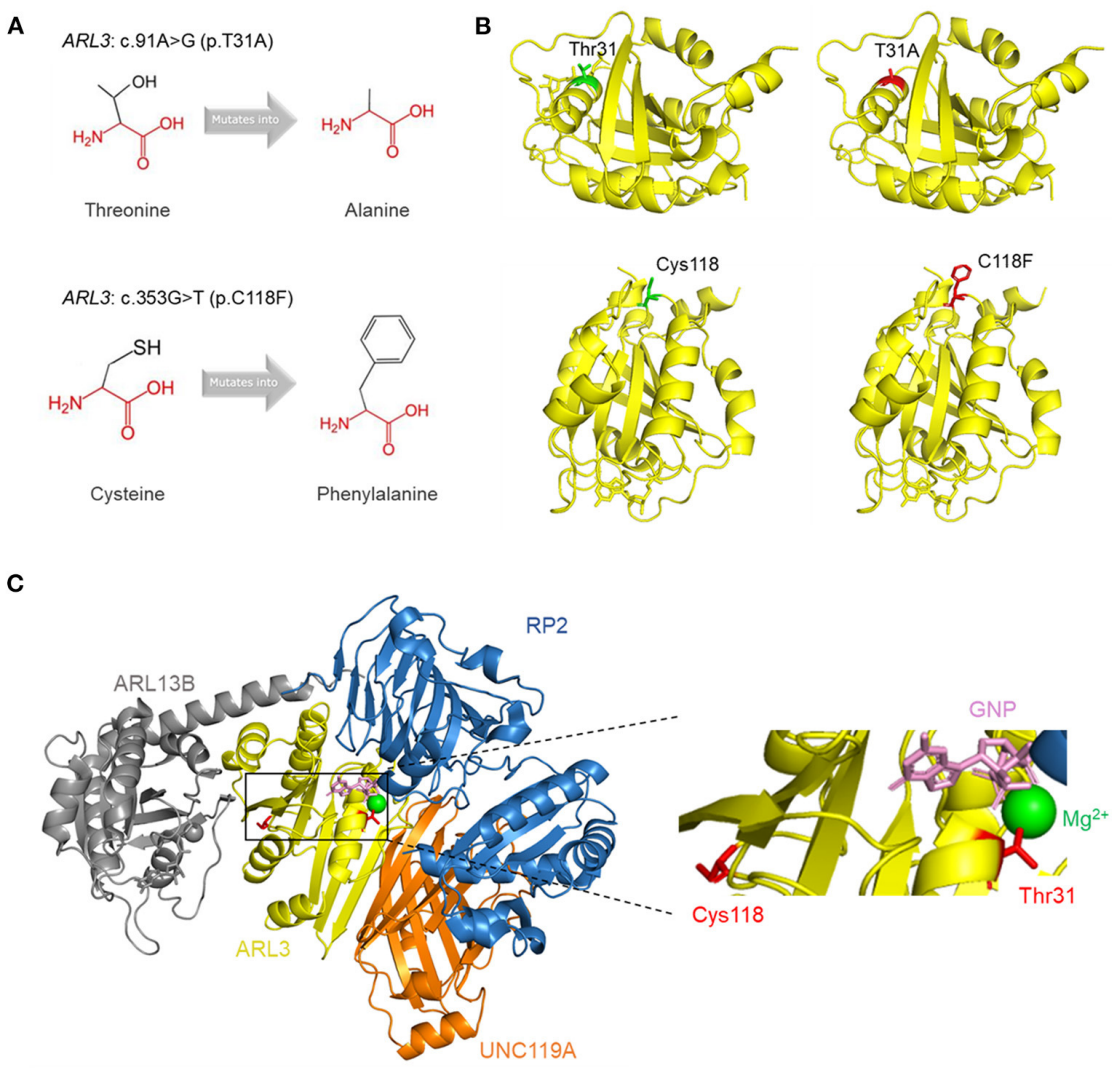
Tertiary structure prediction of mutant proteins. **(A)** The schematic structures of the original and the mutant amino acids. The backbone was colored in red; the side chain was colored in black. **(B)** The 3D-structure of the wild-type and mutant proteins. The main structure of the ARL3 protein was colored in yellow. The side chains of the wild-type and the mutant residues were colored in green and red, respectively. **(C)** Superimposition of the structures of ARL3 (yellow) in complex with its interactors: RP2 (blue; PDB: 3BH6) (Veltel et al., 2008), ARL13B (gray; PDB: 5DI3) (Gotthardt et al., 2015), and UNC119A (orange; PDB: 4GOJ) (Ismail et al., 2012). On the right side is a zoomed-in view of some structures, including GNP (pink), Mg^2+^ (green), and the side chains of Thr31 and Cys118 (red).

To visualize the structure changes of the protein mediated by p.T31A and p.C118F mutations, we used HOPE online software. The schematic structures of the original and the mutant amino acid were shown in Figure 4A. The 3D-structure of wild-type and mutant proteins were shown in Figure 4B. Compared with the wild-type residue, the mutant residue at position 31 was smaller and more hydrophobic, while the mutant residue at position 118 was bigger and more hydrophobic. Among the interactors of ARL3 are RP2, ARL13B, UNC119A, who interact with ARL3 in a GTP dependent manner. In order to visualize the interactions, we superimposed the known structures of ARL3 in complex with the interactors (Figure 4C).

### T31A and C118F Mutations Impaired the Stabilities of ARL3 Proteins

The conformational change of protein caused by the change of amino acid properties was closely related to the stability of protein. Thus, we wondered whether *ARL3* T31A and C118F mutations affected the stability of the protein. The online tools MUpro and I-Mutant v2.0 were used to predict protein stability and results suggested that the two mutant ARL3 proteins showed decrease of stability (Table 2).

**Table 2:** Prediction of the protein stability with online tools.

**Online tools**	**ARL3: c.91A>G (p.T31A)**	**ARL3: c.353G>T (p.C118F)**
Mupro	ΔΔG: −0.19 (DECREASE stability)	ΔΔG: −0.23 (DECREASE stability)
I-Mutant v2.0	ΔΔG: −0.50 (Decrease stability)	ΔΔG: −0.13 (Decrease stability)

*ΔΔG: ΔG (mutated protein) –ΔG (wild type protein) in kcal/mol. ΔΔG <0: decrease stability. ΔΔG > 0: increase stability*.

**Figure 5:**
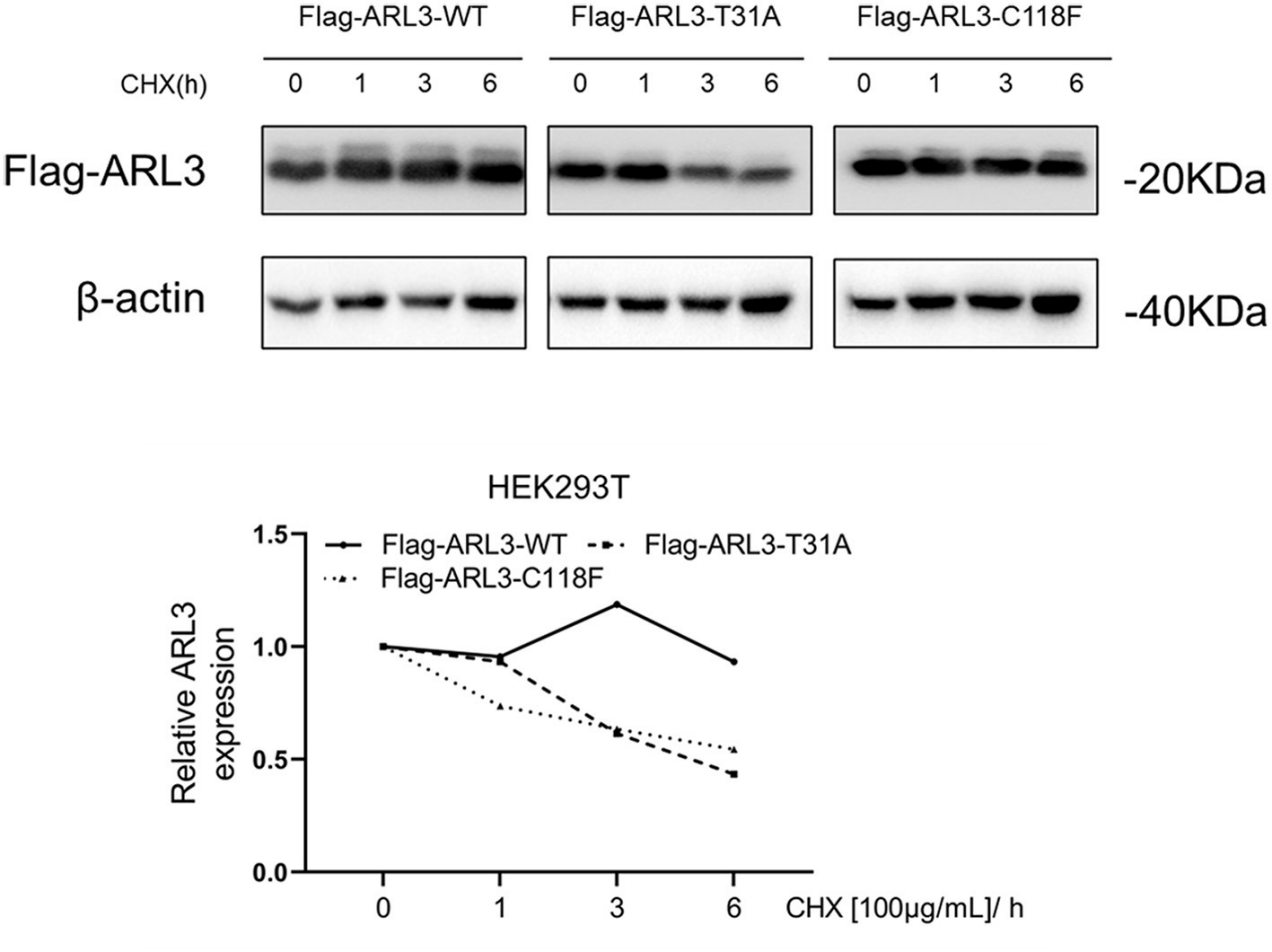
The ARL3 variants T31A and C118F impaired the stabilities of the encoded proteins in HEK293T cells. HEK293T cells were transfected with empty vector, wild-type, *ARL3* T31A and C118F vectors for 24 h and then treated with 100 μg/ml cycloheximide (CHX) for 1, 3, and 6 h, respectively. ARL3 protein levels were detected using the flag antibody. The protein levels of β-actin were used as endogenous control (*n* = 1).

To determine whether *ARL3* T31A and C118F mutations destabilize ARL3 protein, CHX chase assays was performed. HEK293T cells were transfected with empty vector, wild-type and two mutant *ARL3* vectors for 24 h, followed by treatment with cycloheximide (CHX) for 1, 3, and 6 h, respectively. Wild-type and two mutant ARL3 protein levels were analyzed using the flag antibody with Western blot. In Figure 5, both the half-lives of ARL3 T31A and ARL3 C118F protein showed a decreasing trend compared with the wild-type ARL3 protein. These results indicated that both T31A and C118F mutations decreased the stability of ARL3 protein.

### C118F Mutation Disrupted the Protein Interaction With RP2

**Figure 6:**
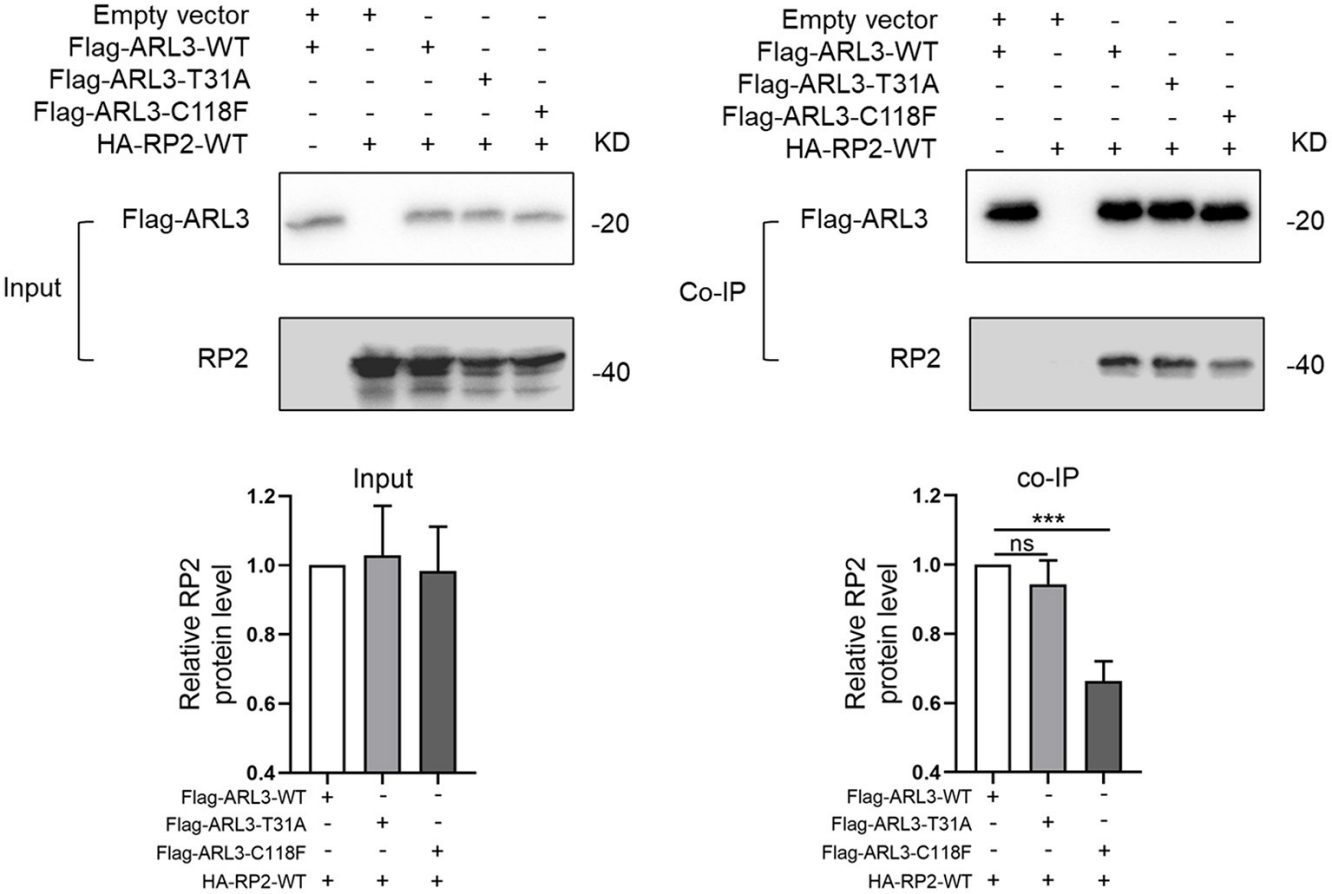
*ARL3* variants T31A and C118F disrupted the combination between ARL3 and RP2. Plasmids of HA-RP2-WT were co-transfected with wild-type or mutant Flag-ARL3 plasmids into HEK293T cells. ARL3 was immunoprecipitated with anti-Flag antibody. Western blotting was performed to detect the specific proteins indicated on the left side of each panel (Error bars indicate means ± SD; *n* = 3, ***p < 0.001).

As a small molecule GTP-binding protein, ARL3 was activated when combined with GTP. But it depends on the presence of functional guanine nucleotide exchange factor, ARL13B (Gotthardt et al., 2015). Protein RP2 could interact with ARL3, and it was identified as a negative regulator by hydrolyzing GTP. In addition, it was reported that the impaired interaction between RP2 and ARL3 induced by RP2 mutation could cause RP (Kuhnel et al., 2006). To investigate whether the novel ARL3 variants affected the interaction between ARL3 and RP2, we conducted co-IP analysis. Plasmid of HA-RP2-WT was co-transfected with Flag-ARL3-WT, Flag-ARL3-T31A, or Flag-ARL3-C118F in HEK293T cells. As in Figure 6, RP2 was detected after Flag-ARL3 pull-down, suggesting interaction between ARL3 and RP2 in HEK293T cells. The relative level of RP2 protein was significantly reduced in the C118F mutant group compared with the control group, while there was no significant change in the T31A mutant group. The results demonstrated that *ARL3* mutation C118F inhibited the interaction between ARL3 and RP2 proteins.

## Discussion

In this study, we identified two novel variants (c.91A>G, p.T31A; c.353G>T, p.C118F) in *ARL3* gene in a Chinese RCD family. According to the American College of Medical Genetics and Genomics (ACMG) standards and guidelines, the two variants were “likely pathogenic,” which was supported by the following evidences. The two variants, (1) were supported by functional tests *in vitro* (PS3); (2) located in an important domain that acts as GTP/Mg2+ binding site and GAP interaction site (PM1); (3) not found in the 200 unrelated health controls and absent from any databases (PM2); (4) The two variants were considered as “Deleterious” by function prediction software, and were highly conserved during evolution (PP3).

The ARL3 Thr31 and Cys118 residues are highly conserved among species, and *in silico* prediction tools suggested that either missense change was likely to be pathogenic. HOPE analysis showed that, compared with the wild-type residues (Threonine and Cysteine), the mutant residue (Alanine) of p.T31A was smaller and more hydrophobic, and the mutant residue (Phenylalanine) of p.C118F was bigger and more hydrophobic. The changes of size and hydrophobicity may cause the loss of hydrogen bonds in the core of the protein, and disturbing protein folding, and then destabilize ARL3 protein. In CHX chase assays, our results revealed that the T31A and C118F protein showed a rapid degradation while the wild-type ARL3 protein was stabilized, indicating the T31A and C118F mutation decreased the stability of ARL3 protein. On the other hand, we noticed that p. Thr31 was central to the P-loop NTPase domain in ARL3, and involved in direct binding of the beta and gamma phosphate moieties of GXP (GTP or GDP) and Mg^2+^ cation (Figure 4C). Another substitution at T31, T31N, has been widely used as a synthetic ARL3-GDP conformational mimic. In addition, the known interaction partners of ARL3 mainly include PDE6D, UNC119A/B, RP2 and ARL13B, whose interactions with ARL3 are GTP-dependent. Therefore, the replacement of Threonine with Arginine at residue 31 variant may impact GTP binding or exchange and thereby affected the normal interactions with these molecules or other parts of the protein, which is most likely a loss-of-function mechanism.

In the mammalian retina, ARL3 functions as a cargo displacement factor and plays an important role in the transport of lipidated protein to the outer segment of the photoreceptors. In addition, RP2 and ARL3 co-localize to the ciliary apparatus (Grayson et al., 2002), and RP2 could stimulate the exchange of GTP to GDP on ARL3, stimulating the release of the lipidated proteins bound to PDE6D and UNC119A/B (Hanke-Gogokhia et al., 2018). On the other hand, Arl3 and RP2 regulated the trafficking of specific ciliary tip kinesins, Kif7 and Kif17, independently of lipidated protein trafficking(Schwarz et al., 2017). The well-regulated protein trafficking in photoreceptors is crucial for normal visual function. The connecting cilium is a key player for the vectorial transport of proteins from the endoplasmic reticulum to the outer segment. Unsurprisingly, mutations in several connecting cilium-associated proteins have been shown to be associated with various types of retinal dystrophy like Joubert syndrome-associated RP, dominant RP, X-linked RP and dominant CRD (Liu et al., 2002; Cantagrel et al., 2008; Thomas et al., 2014; Zhang et al., 2019). It indicates that close cooperation of related proteins is necessary for normal operation of protein transport in photoreceptors.

Our co-IP results revealed that C118F mutant showed a reduced affinity for RP2. But it is unknown how C118F mutant contributes to the occurrence and development of RCD. Significantly, the reported allele frequency of c.353G>T (p.C118F) in GnomAD is concentrated in the East Asian population with almost 1:1,000. This allele frequency is borderline as being too common to cause a very rare disease like RCD. Furthermore, C118F is only conserved in mammals, but not in zebrafish and yeast, and not all the *in silico* prediction tools predicted the C118F variant would be pathogenic. Given that the mother and sister who carried the variant of C118F were unaffected, the mutation C118F may not cause disease when it exists alone as a heterozygote.

On the other hand, when T31A was carried by the father as a heterozygote, he showed a late onset and relatively mild phenotype of CRD. Significantly, the proband who carried the compound heterozygous variants of T31A and C118F typically presented with poor visual acuity, nyctalopia and peripheral field loss when he was young. ERG revealed severely reduced rod and cone system functions. We presumed these two variants may work together to promote the occurrence and development of RCD. Thus, compound heterozygous variants T31A and C118F in *ARL3* caused earlier and severer RCD condition. However, considering the limitation of cell model, the *in vivo* tests of the two variants could not be inferred with confidence at this moment. And it could be helpful to identify whether his father had other gene mutation that caused this phenotype. Further functional studies are still necessary.

**Figure 7:**
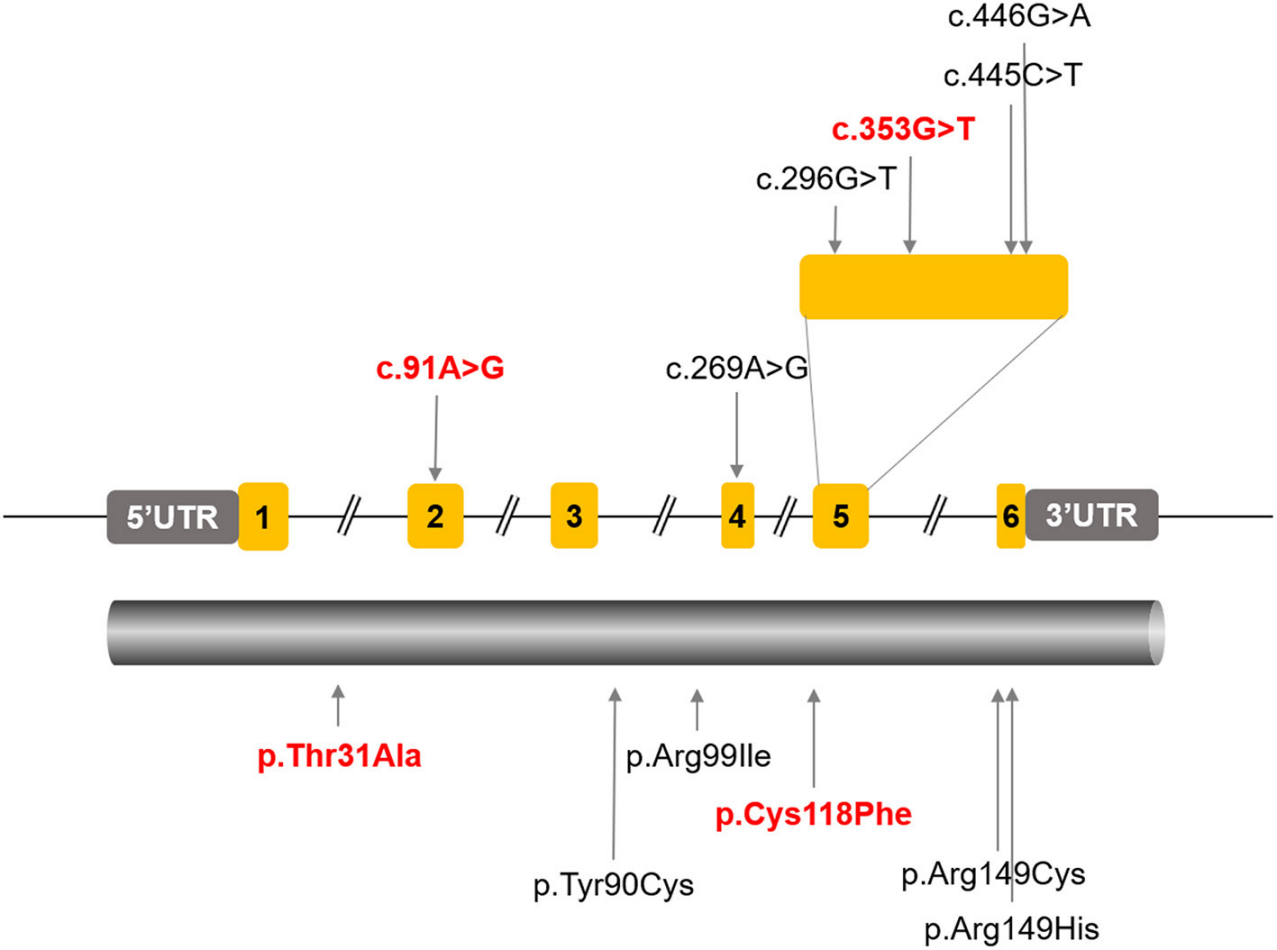
All the reported *ARL3* pathogenic mutations were labeled in the schematic diagram of ARL3 gene and protein. The *ARL3* mutations c.91A>G (p.T31A) and c.353G>T (p.C118F) were indicated in red.

So far, four *ARL3* mutations have been identified (Figure 7), most of which locate at the important domain, affecting the stability of encoded protein or the affinity with interactors. Previously, two different *ARL3* Arg149 missense variants (c.445C>T, p.Arg149Cys; c.446G>A, p.Arg149His) were reported to cause recessive Joubert syndrome, characterized by hypoplasia of the cerebellar vermis, developmental delay, renal anomalies, and RCD in two families (Alkanderi et al., 2018). The study showed that substitution of arginine at position 149 disrupted the known interaction between ARL3 and ARL13B and thus prevented the correct release of intra-ciliary cargos. Patients with heterozygous missense variant (c.269A>G, p.Tyr90Cys) in *ARL3* have recently been found to cause non-syndromic autosomal dominant RP, confirming earlier reports of this missense variant causing retinal disease. The missense variant is predicted to disrupt protein folding and compromise GTP binding or exchange. A most recent report indicated that a homozygous variant in *ARL3* (c.296G>T, p. Arg99Ile) caused CRD in two consanguineous families. The study suggested that the mutation p.Arg99Ile may alter the affinity of encoded protein for guanine nucleotides, resulting in a much less stable protein. Thus, although *ARL3* is not a common cause of retinal degeneration in humans, it is a strong prior candidate gene.

In conclusion, our results extended both genotype and phenotype of *ARL3* associated retinal dystrophy. We defined two novel pathogenic variants (c.91A>G, p.T31A; c.353G>T, p.C118F) of *ARL3* in a Chinese family with typical RCD. We presumed that these compound heterozygous variants were associated with the early onset of recessive RCD, while c.91A>G along might be associated with a late onset of dominant CRD. We further found the *ARL3* variants T31A and C118F destabilized ARL3 protein, and the C118F disrupted the interaction with RP2 in HEK293T cells. On the other hand, considering the limitation of cell model and number of patients, the detailed mechanisms that the two *ARL3* variants causing RCD deserve further studies.

## Data Availability Statement

The datasets presented in this study can be found in online repositories. The names of the repository/repositories and accession number (s) can be found below: https://databases.lovd.nl/shared/individuals?search_owned_by_=%3D%22Leming%20Fu%22.

## Ethics Statement

The studies involving human participants were reviewed and approved by The Ethics Committee of Henan Eye Hospital. Written informed consent to participate in this study was provided by the participants' legal guardian/next of kin. Written informed consent was obtained from the individual(s), and minor(s)' legal guardian/next of kin, for the publication of any potentially identifiable images or data included in this article.

## Author Contributions

BL conceptualized and designed the study. YL, QG, and YY collected the clinical samples and clinical data. LF, YL, and YY performed the genetic analysis and bioinformatics evaluations. LF conducted the molecular biology experiments and drafted the manuscript. BL, SY, and XZ interpreted the results. BL and XZ reviewed and edited the draft. All authors agreed to be accountable for the content of the work and approved the final manuscript.

## Conflict of Interest

The authors declare that the research was conducted in the absence of any commercial or financial relationships that could be construed as a potential conflict of interest.
