# Supplementary material for: Autosomal Recessive Rod-Cone Dystrophy Associated With Compound Heterozygous Variants in ARL3 Gene
Source: Front Cell Dev Biol. 2021 Mar 4;9:635424. doi: 10.3389/fcell.2021.635424 (PMC7969994; doi:10.3389/fcell.2021.635424)
Supplement: Supplementary file 1 [file Data_Sheet_1.docx]

Supplementary Material

# Supplementary Tables

**Supplementary Table 1.** The Primer sequences.

| **Primer** | **Sequence (5’-3’)** |
| --- | --- |
| ARL3-100-F | ATCCTCAGTCTTCTGTTCTCACA |
| ARL3-100-R | ATTTTAACCTTCCCTCCCTCTC |
| ARL3-201-F | AGCACTTCTACCGAAACTTGGA |
| ARL3-201-R | CCATTTTGTCTTTCACATTGCT |

**Supplementary Table 2.** The PCR conditions.

| **Operation** | **Temperature** | **Time** | **Cycles** |
| --- | --- | --- | --- |
| Initial activation | 94℃ | 5 min | 1 |
| Denaturation | 94℃ | 30 sec | 35 |
| Annealing | 50-60℃ | 30 sec |  |
| Elongation | 72℃ | 30-60 sec/kb |  |
| Final elongation | 72℃ | 10 min | 1 |

| **Gene** | **Location** | **Transcription** | **exon** | **cDNA change** | **AA change** | **Zygo-sity** | **PMID** | **ExAC**  **_EAS** | **1000g**  **_EAS** | **gnomAD**  **exome_**  **EAS** | **SIFT** | **Poly-phen**  **-2** | **Muta-tion**  **Taster** | **Condition (s)** |  |
| --- | --- | --- | --- | --- | --- | --- | --- | --- | --- | --- | --- | --- | --- | --- | --- |
| COL9A2 | chr1:40775985 | NM_001852 | 14 | c.689C>T | p.P230L | Het | - | 0.0002 | 0 | 0.0003 | T | D | D | Epiphyseal dysplasia, multiple, 2 (AD);  Stickler syndrome, type V (AR) | |
| USH2A | chr1:215847762 | NM_206933 | 63 | c.13491T>A | p.F4497L | Het | - | 0 | 0 | 0 | T | B | N | Retinitis pigmentosa 39 (AR); Usher syndrome, type 2A (AR) | |
| USH2A | chr1:215933036 | NM_206933 | 57 | c.11197A>G | p.N3733D | Het | - | 0 | 0 | 0 | T | B | N | Retinitis pigmentosa 39 (AR); Usher syndrome, type 2A (AR) | |
| CNGA3 | chr2:99006142 | NM_001079878 | 5 | c.T417G | p.D139E | Het | - | 0.0005 | 0.002 | 0.0008 | T | B | N | Achromatopsia 2 (AR) | |
| ATXN7 | chr3:63898360 | NM_000333 | 3 | c.86_87insGCA | p.R29  delinsRQ | Het | - | 0 | 0 | 0.0556 | - | - | - | Spinocerebellar ataxia 7 (AD) | |
| WDR19 | chr4:39233878 | NM_001317924 | 18 | c.1759A>G | p.I587V | Het | - | 0.0068 | 0.0109 | 0.0067 | T | B | N | Cranioectodermal dysplasia 4 (AR); Nephronophthisis 13 (AR); Senior-Loken syndrome 8 (AR); Short-rib thoracic dysplasia 5 with or without polydactyly (AR) | |
| FBN2 | chr5:127697538 | NM_001999 | 19 | c.2432T>C | p.I811T | Het | - | 0 | 0 | 0 | D | D | D | Contractural arachnodactyly, congenital (AD); Macular degeneration, early-onset (AD) | |
| RP1L1 | chr8:10467637 | NM_178857 | 4 | c.3971A>G | p.E1324G | Het | - | 0 | 0 | 0 | T | B | P | Occult macular dystrophy (AD) | |
| RP1L1 | chr8:10467652 | NM_178857 | 4 | c.3956C>G | p.A1319G | Het | - | 0.0002 | 0 | 0 | T | B | N | Occult macular dystrophy (AD) | |
| RP1 | chr8:55533945 | NM_006269 | 2 | c.419A>C | p.H140P | Het | - | 0 | 0 | 0 | T | B | N | Retinitis pigmentosa 1 (AR, AD) | |
| VPS13B | chr8:100887784 | NM_017890 | 62 | c.11959C>G | p.P3987A | Het | - | 0.0101 | 0.0159 | 0.0114 | T | B | D | Cohen syndrome (AR) | |
| RECQL4 | chr8:145738080 | NM_004260 | 17 | c.2830C>T | p.H944Y | Het | - | 0.0001 | 0.001 | 0.0001 | - | B | - | Baller-Gerold syndrome (AR); RAPADILINO syndrome (AR); Rothmund-Thomson syndrome (AR) | |
| POLG | chr15:89869955 | NM_001126131 | 9 | c.1600A>G | p.S534G | Het | - | 0.0001 | 0.001 | 0.0003 | T | B | D | Mitochondrial DNA depletion syndrome 4A (Alpers type) (AR); Mitochondrial DNA depletion syndrome 4B (MNGIE type), (AR); Mitochondrial recessive ataxia syndrome (includes SANDO and SCAE), (AR); Progressive external ophthalmoplegia, (AD, AR) | |
| RPGRIP1L | chr16:53670426 | NM_015272 | 22 | c.3221-2A>G | - | Het | - | 0.0002 | 0.001 | 0.0001 | - | - | D | COACH syndrome (AR); Joubert syndrome 7 (AR); Meckel syndrome 5 (AR) | |
| MKS1 | chr17:56291147 | NM_017777 | 7 | c.728C>T | p.T243M | Het | - | 0.0001 | 0 | 0.00005798 | T | B | N | Bardet-Biedl syndrome 13 (AR); Joubert syndrome 28 (AR); Meckel syndrome 1 (AR) | |

**Supplementary Table 3.** Other detected mutations carried by the proband.

**Supplementary Figures**


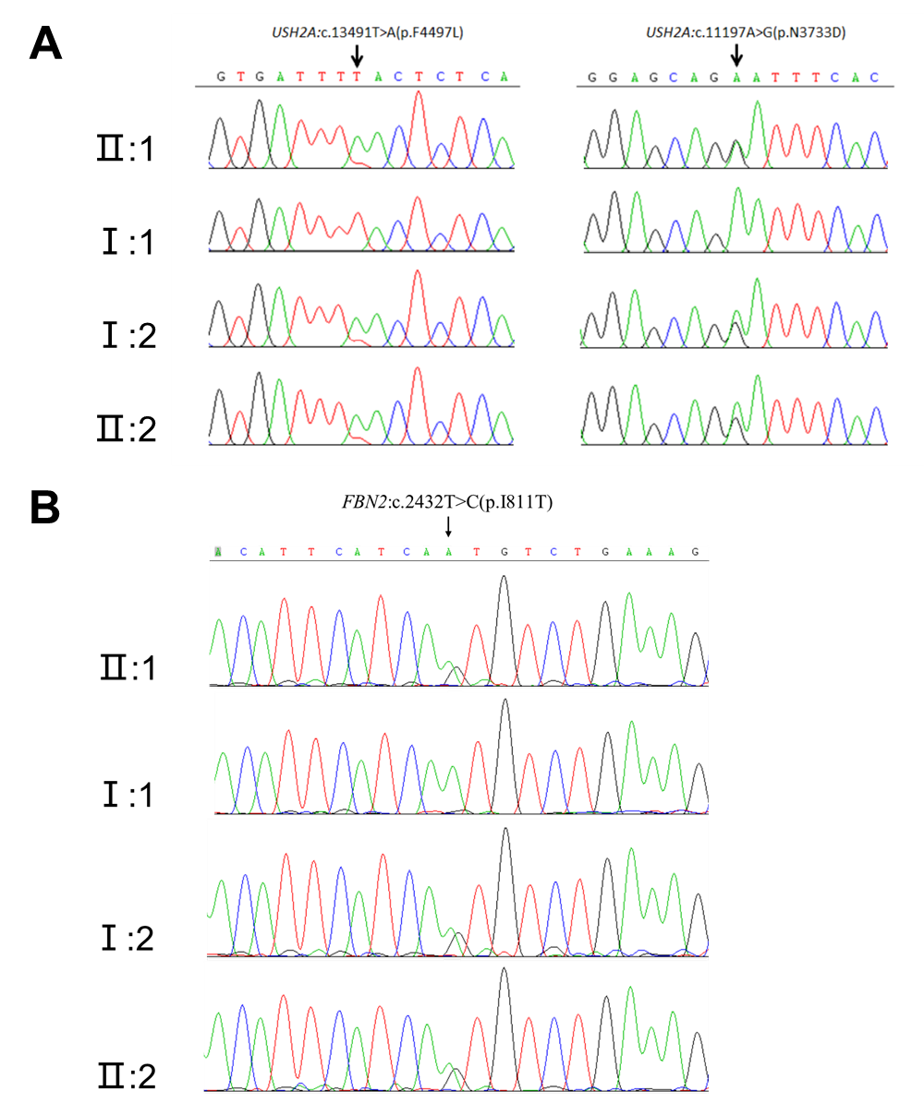


**Supplementary Figure 1.** Sanger sequencing for two heterozygous missense variants (c.13491T>A, p.F4497L; c.11197A>G, p.N3733D) in *USH2A* and a heterozygous missense variant (c.2432T>C, p.I811T) in *FBN2* in all family numbers.
